# Smell the change: On the potential of gas‐chromatographic ion mobility spectrometry in ecosystem monitoring

**DOI:** 10.1002/ece3.3990

**Published:** 2018-04-02

**Authors:** Wolfgang Vautz, Chandrasekhara Hariharan, Maximilian Weigend

**Affiliations:** ^1^ Leibniz‐Institut für Analytische Wissenschaften – ISAS – e.V. Dortmund Germany; ^2^ ION‐GAS GmbH Dortmund Germany; ^3^ Nees‐Institut für Biodiversität der Pflanzen Bonn Germany

**Keywords:** biodiversity, gas‐chromatography, ion mobility spectrometry, monitoring, plant metabolites, volatile organic compounds

## Abstract

Plant volatile organic compounds (pVOCs) are being recognized as an important factor in plant–environment interactions. Both the type and amount of the emissions appear to be heavily affected by climate change. A range of studies therefore has been directed toward understanding pVOC emissions, mostly under laboratory conditions (branch/leaf enclosure). However, there is a lack of rapid, sensitive, and selective analytical methods, and therefore, only little is known about VOC emissions under natural, outdoor conditions. An increased sensitivity and the identification of taxon‐specific patterns could turn VOC analysis into a powerful tool for the monitoring of atmospheric chemistry, ecosystems, and biodiversity, with far‐reaching relevance to the impact of climate change on pVOCs and vice versa. This study for the first time investigates the potential of ion mobility spectrometry coupled to gas‐chromatographic preseparation (GC‐IMS) to dramatically increase sensitivity and selectivity for continuous monitoring of pVOCs and to discriminate contributing plant taxa and their phenology. Leaf volatiles were analyzed for nine different common herbaceous plants from Germany. Each plant turned out to have a characteristic metabolite pattern. pVOC patterns in the field would thus reflect the composition of the vegetation, but also phenology (with herbaceous and deciduous plants contributing according to season). The technique investigated here simultaneously enables the identification and quantification of substances characteristic for environmental pollution such as industrial and traffic emissions or pesticides. GC‐IMS thus has an enormous potential to provide a broad range of data on ecosystem function. This approach with near‐continues measurements in the real plant communities could provide crucial insights on pVOC‐level emissions and their relation to climate and phenology and thus provide a sound basis for modeling climate change scenarios including pVOC emissions.

## 1 INTRODUCTION

The monitoring of biodiversity and of ecosystem status and change have become major scientific disciplines in the last few years (Pocock et al., 2015). Climate change is considered a major factor of ecosystem change and causes a broad range of physiological and ecological changes, including species displacements and migrations. Shifts in ecosystem composition and function in turn are known to influence the emission of plant volatile organic compounds (pVOCs, Lahr, Schade, Crossett, & Watson, 2015; Constable, Guenther, Schimel, & Monson, 1999; Constable, Litvak, Greenberg, & Monson, 1999; Pegoraro, Rey, Abrell, Vanharen, & Lin, 2006; Purves, Capsersen, Moorcroft, Hurtt, & Pacala, 2004; Šimpraga et al., 2011). Emissions are notably influenced by herbivore attacks, drought, and temperature (Pegoraro et al., 2006; Seco et al., 2015) and are thus an excellent indicator of stress for an ecosystem, providing a real‐time early warning system for shifting conditions, tipping points, and ecosystem change. Plant volatile organic compounds (pVOCs) are in turn increasingly recognized as part of complex feedback processes between vegetation and atmosphere (Peñuelas, Rutishauser, & Filella, 2009; Peñuelas & Staudt, 2010; Pryor, Hornsby, & Novick, 2014), with an overall positive feedback of increasing pVOC concentrations on temperature and ozone concentrations (Peñuelas & Staudt, 2010). Due to the importance of an in‐depth understanding of plant volatile organic compound emissions, a wide range of studies both at the species level (Blande, Tiiva, Oksanen, & Holopainen, 2007; Burkle & Runyon, 2016; Constable, Guenther, et al., 1999; Constable, Litvak, et al., 1999; Lee & Seo, 2014) and from the perspective of entire ecosystems (Lahr et al., 2015; Purves et al., 2004; Seco et al., 2015; Wang, Owen, Li, & Peñuelas, 2007) have been published. From those studies, it was also shown by preconcentration on SPME (solid phase micro extraction) and subsequent GC‐MS laboratory analysis, that even groundwater pollution could influence the emissions of plants due to volatilization of contaminants (Mothes, Reiche, Fiedler, Moeder, & Borsdorf, 2010; Reiche, Lorenz, & Borsdorf, 2010).

In spite of these studies, however, major knowledge gaps persist, which are mainly due to the technical limitations of the available methodologies. Most research on pVOCs is conducted with branch/leaf enclosure experiments (Materić et al., 2015), providing accurate measurements of the quantity and quality of the pVOCs (Šimpraga et al., 2011). For calculating vegetation level emissions, the data have to be extrapolated based on known (species specific) pVOC emissions per unit dry leaf mass in combination with estimates of overall leaf mass per unit area (Lahr et al., 2015; Purves et al., 2004), introducing a range of uncertainties into the calculations. Alternatively, the ambient air can be analyzed directly (Seco et al., 2015), but here the relative and absolute contribution of individual plant species tend to remain unclear and analyses frequently have to be restricted to a subset of chemical substances for methodological reasons, typically isoprenes (Pegoraro et al., 2006; Seco et al., 2015).

However, in order to reliably model vegetation level changes in pVOC emissions, changes across the different (major) species components have to be recorded in long time series of more or less continuous measurements. However, the commonly used, very expensive, and large mass spectrometry instruments are usually stationary and can be used for individual measurements only. Furthermore, monitoring different climatic (temperature, humidity) and ecological factors (herbivore attack, shifts in species composition) as well as the concomitant pVOC‐loads in the ambient air is required, ideally in parallel with other sources of atmospheric chemistry (pollutants such as pesticides). pVOC emissions in temperate regions are expected to be strongly affected by phenology, for example, by bud break, anthesis, fruit maturation, and leaf fall (Peñuelas & Staudt, 2010). However, no data sets on real‐life shifts in pVOC emissions in response to phenology have been provided for temperate vegetation—mainly because of the technical limitations currently in place.

pVOC measurements presently have been carried out mostly in a laboratory setups, using preconcentration tools and subsequent thermal desorption for gas‐chromatography coupled to mass spectrometry (GC/MS) (Tholl et al., 2006). GC/MS analysis is comprehensive and—by using preconcentrations steps—also sensitive. However, the instruments are bulky and expensive, sampling, and analyses are time‐consuming, and therefore, GC/MS is not suitable for continuous or even quasi‐continuous monitoring. Other methods applied for a fast analysis such as electronic noses (e.g., Kunert, Biedermann, Koch, & Boland, 2002) are not sufficiently selective to provide rapid and concurrently sensitive, selective pVOC identification and quantification.

Accordingly, there is a pressing need of tools for continuous monitoring of ecosystem function and associated responses of biodiversity as well as for the relevant environmental parameters. In this context, for the first time we investigated the potential of ion mobility spectrometry (IMS) coupled to gas‐chromatographic (GC) preseparation for a continuous monitoring of biodiversity. In particular, we used gas‐chromatographic preseparation (GC‐IMS) for the characterization of species‐specific metabolite patterns, potentially opening the way to identify plant species in a natural community and quantify their phenology.

## 2 ION MOBILITY SPECTROMETRY COUPLED TO GC‐IMS

Ion mobility spectrometry is an analytical method for the sensitive and selective identification and quantification of trace substances in the gas phase. In general, the operational gas (nitrogen or synthetic air)—the so‐called drift gas—is ionized by a radioactive source which initially ionizes the nitrogen molecules. After a multistep reaction chain, protonated water clusters remain as stable reactant ions—the full theory is described in the literature (Eiceman, Karpas, & Hill, 2016). The ions are accelerated in a weak electric field (330 V/cm) toward the Faraday plate functioning as detector. The drift region is separated from the ionization region by a Bradbury–Nielsen grid. This grid is opened every 100 ms for 100 μs to allow an ion cloud to enter the drift region. During their drift toward the detector, the ions collide with the present drift gas molecules. Collision frequency depends on the shape and size of the ion and results in a characteristic resulting velocity of the ion in the drift region. Measuring the drift time of the ion and normalizing it to drift length, temperature, and pressure permits the calculation of the reduced ion mobility, which is characteristic for the ion (Vautz, Bödecker, Bader, & Perl, 2009).

When a sample containing a particular analyte with a proton affinity higher than water is introduced in the ionization region, proton transfer from the reactant ions to the analyte molecules takes place. Those ions in general have different collision cross sections and therefore reach the detector with a different velocity which is characteristic for the analyte and renders its identification possible.

Starting in the 1970s, this technology was first applied for military purpose such as the detection of chemical warfare agents and later on for security, for example, at airports for the detection of drugs and explosives (Eiceman et al., 2016). However, those instruments are optimized to detect only the targeted compound groups and therefore do not provide the full analytical performance. In the last two decades, IMS research has focused more and more on other, civilian applications in process control (Baumbach, 2006; Vautz, Mauntz, Engell, & Baumbach, 2009), biology (Perl et al., 2011; Vautz & Baumbach, 2008), and medicine (Perl et al., 2009; Vautz et al., 2010).

All these applications have in common, that the samples are extremely complex mixtures, often with high humidity. Introducing such a sample directly into the ionization region leads to clustering of the different ions with each other and with water molecules, thus complicating identification or rendering it impossible. To solve this problem, IMS is coupled to rapid gas‐chromatographic preseparation. Thereby, analytes ideally reach the ionization region already preseparated, thus avoiding the clustering effect on the one hand and additionally obtaining characteristic retention times as a measure for identification on the other.

**Figure 1:**
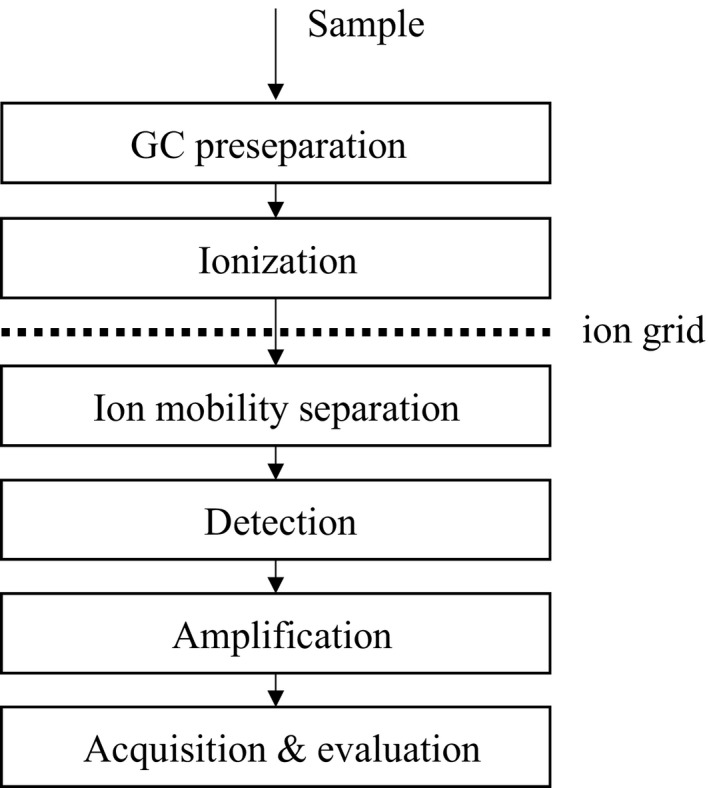
Schematic presentation of the functionality of a GC‐IMS

The functional scheme of an IMS measurement is shown in Figure 1. The experimental setup required is quite simple—the method works at ambient pressure and temperature. Therefore, small and even mobile instruments are available today without compromising the analytical performance. This makes the method highly relevant for many applications where in situ identification of volatile compounds is required.

It needs to be emphasized, that under field conditions with fluctuating temperature and pressure, the performance of the instrument is still reliable. This is due to the fact, that the reduced ion mobility obtained from the measurements is the inverse of the measured drift time of the ions normalized to the experimental (electric field and drift length) and to the environmental conditions (temperature and pressure). Therefore, the reduced ion mobility is stable under normal environmental conditions. Furthermore, with the reactant ion peak, the instruments provide an internal standard for the determination/validation of the mobility values.

In the complex data obtained from such measurements consists of signal intensity vs. ion mobility and retention time. Particular constituents of the sample can be identified by comparing those values for the peaks detected with a reference data base (Bödeker, Vautz, & Baumbach, 2008; Perl, Bödeker, Jünger, Nolte, & Vautz, 2010). If unknown peaks are detected, parallel measurements on adsorption materials and subsequent thermal desorption GC/MS analysis provide a hypothesis on their identity, which can then be validated by GC/IMS analysis of the respective reference substances and inclusion in the reference database. For quantification, the instruments are calibrated by evaporation of the reference analyte and dilution down to the ppt_V_ range by help of a suitable calibration gas generator (Vautz & Schmäh, 2009).

## 3 EXPERIMENTAL

**Figure 2:**
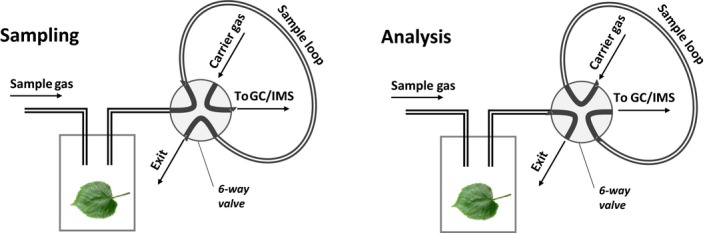
Scheme of the experimental setup for detecting the volatiles from leaves. During sampling (left), the sample loop is flushed with the headspace of the leaves. During analysis (right), the volume of the sample loop is introduced into the analytical system

For the determination of the pattern of volatiles released from the leaves, they were placed in a 20‐ml glass vial and the headspace was introduced in the GC‐IMS system as summarized in the scheme in Figure 2. The vial with the leaves was flushed with a continuous sample flow (100 ml/min) to enable a stable equilibrium. For sampling, the headspace of the leaves is flushed by the sample flow through the sample loop with a volume of 8 ml. For analysis, the 6‐way valve is switched and now the volume of the sample loop is introduced into analytical system consisting of the GC preseparation column followed by the ion mobility spectrometer.

### 3.1 GC‐IMS

The GC‐IMS used for the present experiments was custom‐made at *Leibniz‐Institut für Analytische Wissenschaften*—*ISAS*—*e.V*., Dortmund Germany. It consists of an 8‐ml Teflon sample loop, a 20‐cm multi‐capillary GC column with OV‐5 coating operated at constant 40°C for gas‐chromatographic preseparation (Sibertech, Novosibirsk, Russia), a 500 MBq Ni^63^ ß‐radiation source for ionization and a 12‐cm drift region operated at 330 V/cm. The drift region was separated from the ionization region by a Bradbury–Nielsen grid, and the ions were detected by a Faraday plate. The IMS was operated under ambient conditions with regard to temperature and pressure, and the sample loop was operated at 40°C to avoid memory effects.

Data acquisition software was also custom‐made at ISAS, and the data processing and evaluation was carried out by IONysos, a custom‐made software by ION‐GAS GmbH, Dortmund, Germany. In particular, the software automatically normalizes the drift time to the known ion mobility of the reactant ion peak (Vautz, Bödecker, et al., 2009) and the retention time to that of a known compound (Bödeker et al., 2008; Perl et al., 2010). After the automatic or manual location of a particular compound peak (ion mobility and rentention time), signal intensities, and peak volumes can be compared for a vast data set. Moreover, after a previous calibration, signal intensities can be converted into concentrations (Vautz & Schmäh, 2009). Finally, regions of interest can be defined and a weight can be assigned related to the relevance of the particular signal, thus enabling an automatic characterization of an unknown data set later on.

### 3.2 Headspace sampling

For the analysis, 3 g of the leaves of each plant was introduced into a 20‐ml glass vial at 60°C. This was performed immediately after harvesting the leaves, and the leaves were placed in the vial as a whole. The glass vial was flushed with a carrier gas flow of 100 ml/min nitrogen (quality 6.0) and after 5 min—to enable a steady state in the vial—the carrier gas was introduced into the sample loop of the GC‐IMS for 10 s to avoid memory effects.

### 3.3 Exemplary plants

In order to investigate the presence of taxon‐specific differences in pVOC emissions, we selected a total of nine common herbaceous plant species from Central Europe (see Table 1). Some of them have a characteristic vegetative odor detectable by the human nose (*Cota tinctoria, Lamium album*) and thus were expected to have a characteristic “bouquet” detectable by the analytical technique of GC‐IMS here employed. However, the other plants investigated were specifically chosen because they have no distinctive scent at all (e.g., *Carex muricata, Juncus effuses, Galium album*) to evaluate the potential of the method also for such species.

**Table 1:** Exemplary herbaceous plant species from Central Europe with abbreviations used

CM	*Carex muricata*
CT	*Cota tinctoria*
GA	*Galium album*
LS	*Lathyrus sylvestris*
JE	*Juncus effusus*
LA	*Lamium album*
JM	*Jasione montana*
SF	*Silene flos‐cuculi*
SV	*Silene viscaria*

### 3.4 Data acquisition and evaluation, reproducibility

Before each particular measurement, a blank with clean and humid nitrogen was measured to avoid memory effects. We repeated the measurements for three different plants of each species and for each particular plant at least three times with different leaves. Data were acquired using qIMS software (ISAS, Dortmund, Germany), and data evaluation was carried out using IONysos (ION‐GAS GmbH, Dortmund, Germany).

In general, when starting investigations on particular plants or ecosystems, respectively, some of the detected peaks will not be included in the GC‐IMS substance database. In this case, reference analysis of the samples drawn on adsorption materials and later thermal desorption GC‐MS will be carried out for a comprehensive analysis. By comparison of the GC/IMS and GC‐MS retention time, proposals for the identity of the unknown substances can be obtained and will be validated subsequently by analysis of the pure substance. From this procedure, the completeness of the substance database will increase with time and will render the identification of more on more substances directly from the GC‐IMS measurements possible.

This procedure was not carried out for the present study due to the high technical expenditure and the required personnel. However, from the procedure chosen here, it is assured that the compounds detected derive from the plants only. But certainly, for application of the method in an outdoor scenario, all the particular analytes detected would need to be identified to enable their classification as pVOCs, pesticides, or industrial/traffic pollutants.

**Figure 3:**
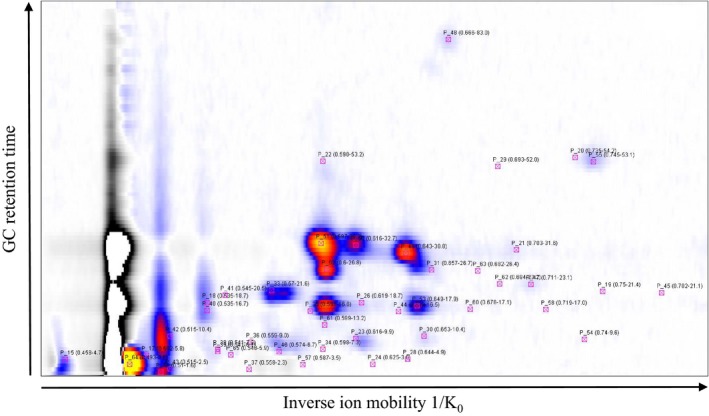
GC‐IMS chromatogram of the headspace of CT leaves presented as color coded signal intensities vs. inverse ion mobility (*x*‐axes) and retention time (*y*‐axes). All peaks found as relevant for one or more plants in this study are indicated

In the following, GC‐IMS data are presented as three‐dimensional chromatogram with the inverse ion mobility as *x*‐axes (which is proportional to the measured drift time), with the retention time as *y*‐axes and the color coded signal intensity (see Figure 3 as an example).

## 4 RESULTS AND DISCUSSION

In the first step, leaves of all particular plants were analyzed as described above. The experiments were repeated three times to validate reproducibility of the results. The GC‐IMS chromatogram—presenting color coded signal intensities vs. inverse ion mobility and retention time—is shown in Figure 3 for CT as an example. All signals investigated for the entire set of plants are indicated, even if they are not present in the metabolites of this specific plant. In total, we found 47 peaks occurring in the headspace of the leaves of one or more plants.

**Figure 4:**
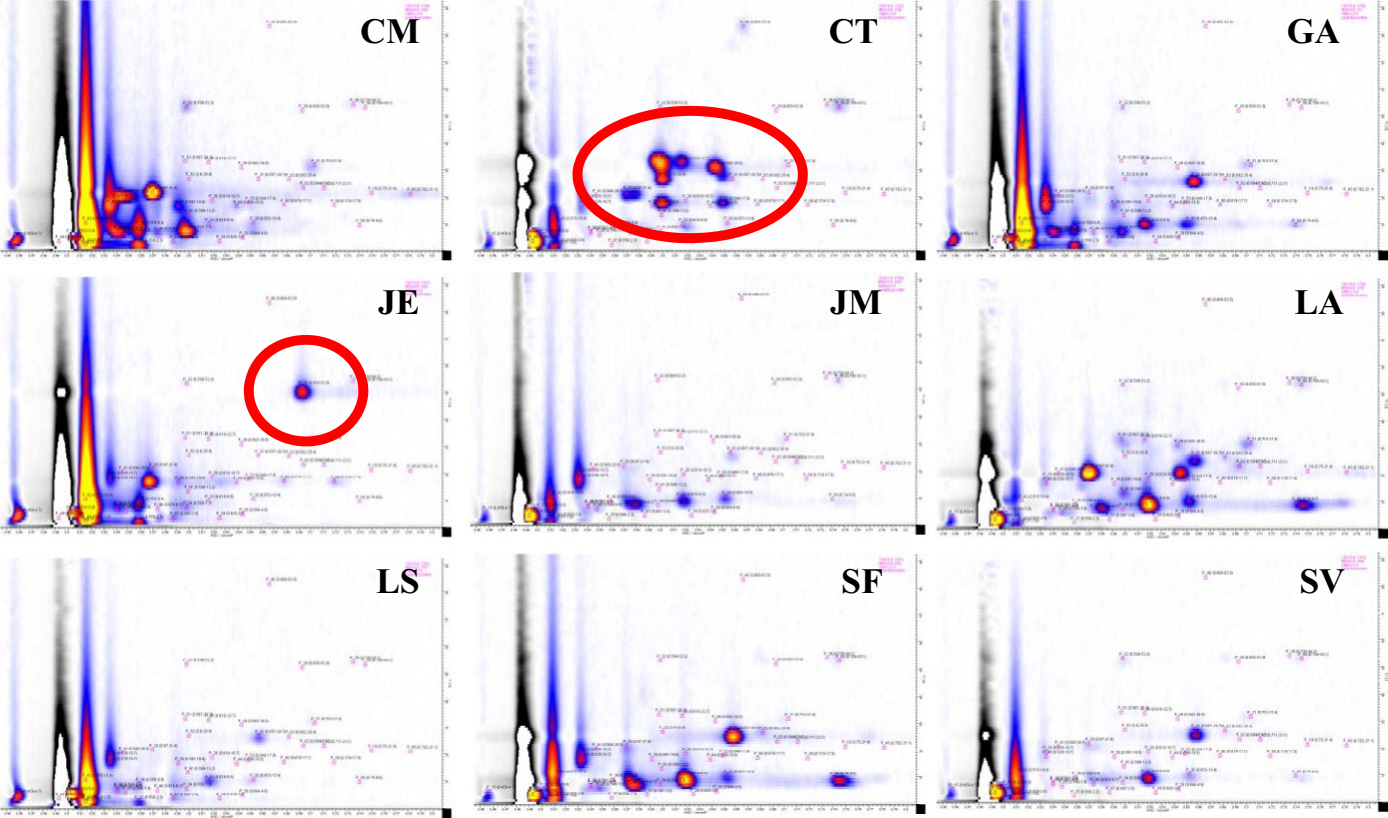
GC‐IMS chromatograms of all nine specific plants investigated. The characteristic pattern for CT and the unique signal found for JE are indicated as an example for the obvious differences between the particular chromatograms

From the analysis of the leaves headspace for CT, a significant pattern of intense peaks can be seen as a prominent circle of red to yellowish dots in Figure 3. This pattern is unique compared to the patterns of the other plants as indicated by a red circle in Figure 4, which presents the characteristic GC‐IMS chromatograms for the nine different plants. The same is true for an unique signal found for JE, which is also indicated by a red circle in Figure 4.

**Figure 5:**
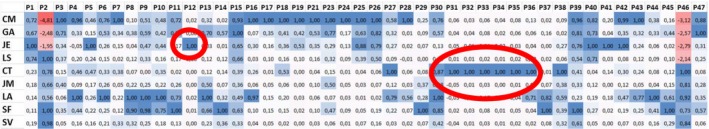
Signal intensities (dark grey = high intensity) of all 47 peaks investigated for all nine specific plants. The particular pattern found for CT and the unique peak found for JE are indicated

Already from a visual inspection, one can notice that each plant has characteristic peaks or patterns of peaks. However, to enable an objective comparison of the data, peak intensities of all peaks investigated the signal intensities of all 47 peaks and all nine plants are presented in a grayscale coded table in Figure 5. In this way, the intensities of all peaks detected as relevant can be converted to a kind of “barcodes” of the specific plants. This table reflects the characteristic patters—see the big circle for the CT pattern and the smaller circle indicating the unique peak of JE as examples. These are the most obvious examples for the characteristic features of the detected VOC patterns. However, it is possible to develop an algorithm based on the detected pVOCs, which enables a definitive identification of each of the plants out of the collective investigated here.

**Figure 6:**
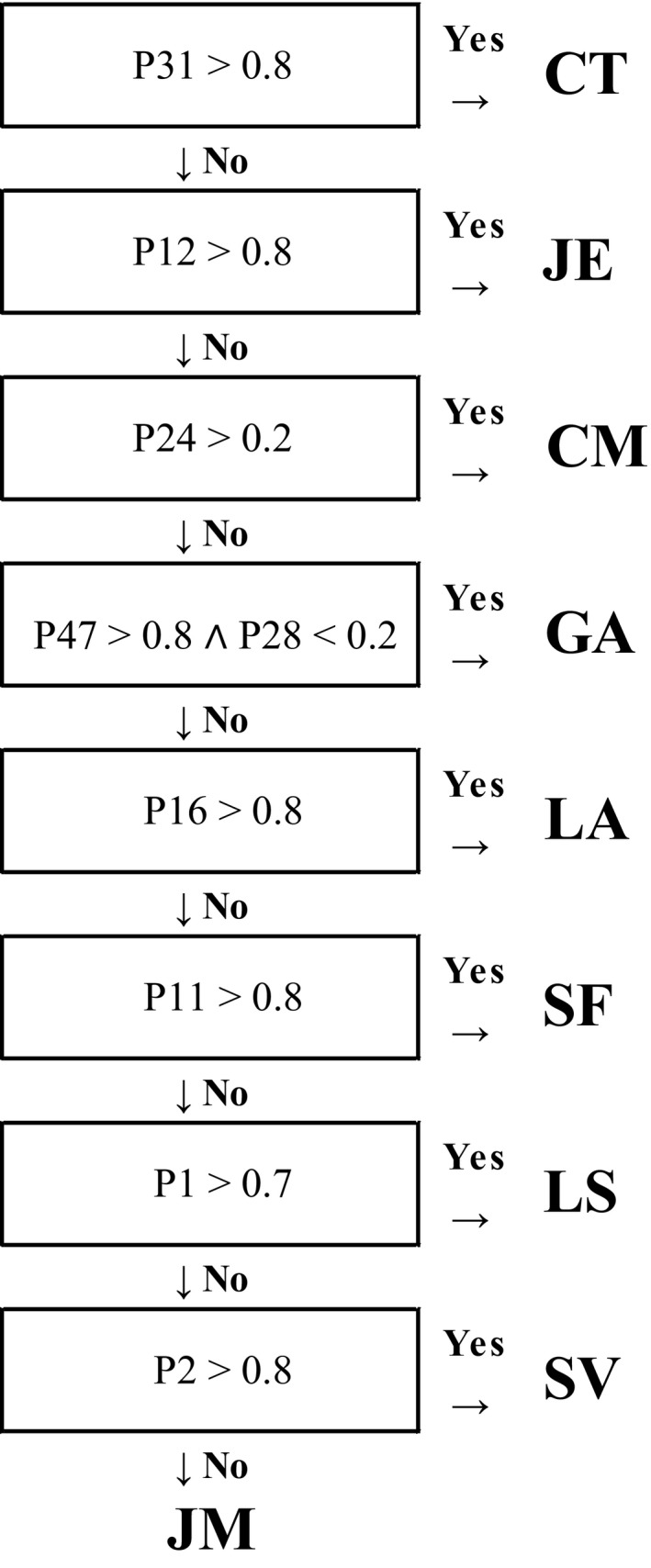
Decision tree based on nine detected signals allowing a definite identification of each of the plants of the collective

It is obvious that the pattern of 47 peaks is much more complex than required for a reliable differentiation of the nine plant species. Therefore, we condensed the table in Figure 5 the minimum number of peaks required for identification of the specific plants using the most significant signals first. Furthermore, we used threshold values for each of the peaks. By that, we can built a decision tree as demonstrated in Figure 6, which enables the identification of each one of the plants from on nine peaks detected in the headspace as describes above.

## 5 CONCLUSIONS AND OUTLOOK

We here demonstrate that a unique and characteristic pattern of metabolites could be determined for each specific plant in the set of nine species investigated here—even those which have no perceptible odor to the human nose—using ion mobility spectrometry with gas‐chromatographic preseparation (GC‐IMS) under the experimental conditions described. The present experiment serves as a proof‐of‐concept—the minute amounts of volatiles produced by the plants can be efficiently detected and provide species‐specific patterns. Future studies should clearly address shifts in volatile emission in relation to phenology. The data provided here clearly indicate that GC‐IMS could be applied in the field for monitoring patterns in a plant community to detect changes in species composition and phenology. For a field monitoring, the characteristic metabolite patterns need to be referenced in a comprehensive database, built up by laboratory measurements from vouchered plants in cultivation, to provide a solid standard. The technique used here is able to monitor pVOCs in a quais‐continuous way (in approx. 10 min interval), providing the potential to continuously monitor volatile emissions more or less in real time, that is, faster than plants are likely to adjust their emissions to changes in humidity, radiation, or temperature. Thus, GC‐IMS permits a very close monitoring of pVOC emissions in relation to all biotic and abiotic factors.

Under real‐life atmospheric conditions pVOCs concentrations can be expected to be much lower than in the setup chosen here. Therefore, further work has to be directed toward significant increase in sensitivity, for example, using in‐line preconcentration tools. The sensitivity of the technique in the present setup is in the low ppb_V_ down to the ppt_V_ range. However, in‐line preconcentration tools can be applied and, with sampling intervals of few minutes, detection limits in the ppq_V_ range can be obtained. This work is already in progress at ISAS, Dortmund, Germany. With such a setup, the concentration range to be expected in nature can be covered in a quasi‐continuous mode with a temporal resolution below 1 h.

Furthermore, a comprehensive database of pVOCs—probably focused on a particular ecosystem—needs to be developed, including anthropogenic compounds such as pesticides and pollutants from industry or traffic. For this reason, all compounds detected need to be identified to permit their classification as pVOCs, pesticides, or industrial/traffic pollutants.

In the present study, by comparison with our substance data base, several compounds could be identified such as many terpenes (e.g., 3‐Carene, Limonene, γ‐Terpinene), compounds usually found in essential oils and some aldehydes could be identified. However, those results have not been independently validated. Other compounds not included in the database need to be identified in the future. To do so, additional GC‐MS measurements are required, followed by a validation with reference compounds. This has to be performed only once and will permit automatic identification in the following. Moreover, the pVOC patterns documented here will also need to be investigated with regard to stability, phenological variation, and to environmental and anthropogeneous influences.

With the data provided by this method, GC‐IMS has the potential to give additional and in particular continuous information on short‐term and long‐term variations in pVOC emissions, permitting detailed insights into the physiological status of vegetation, its reaction to changing environmental conditions, and the feedback between vegetation and climate. In real‐world applications, the patterns recorded are likely to be more complex and at this stage it will be only possible to determine the major components (dominant species) for a given ecosystem, plus the major contaminants. However, these data will already provide a very good proxy of ecosystem reactions to intrinsic and extrinsic factors and permit a new approach in ecosystem modeling and the quantification of ecosystem services.

The aim of the presented approach is thus not the complete documentation of biodiversity in a given habitat—which will not change in minutes or hours—but rather to trace physiological ecosystem reactions in near real‐time and thus to understand ecosystem reaction to extrinsic and intrinsic factors. These physiological shifts are what precedes and often causes shifts in ecosystem function and biodiversity. It is exactly these shifts that have been impossible to detect and record with any precision in the past, and it is these shifts we need to understand in order to monitor and model ecosystem reactions to, for example, climate change. Furthermore, there is potential for the monitoring of many other compounds relevant for ambient air chemistry and thus on climate.

## CONFLICT OF INTEREST

All authors disclose any potential sources of any conflict of interest.

## AUTHORS CONTRIBUTION

W. Vautz and M. Weigend developed the idea for this research. M Weigend chose and provided the particular plants. C. Hariharan carried out the measurements based on the experimental setup developed together with W. Vautz. C. Hariharan and W. Vautz evaluated and interpreted the data. All authors discussed the results and contributed to the final manuscript.
